# High proportion of transient neonatal zinc deficiency causing alleles in the general population

**DOI:** 10.1111/jcmm.13982

**Published:** 2018-11-18

**Authors:** Yarden Golan, Adrian Lehvy, Guy Horev, Yehuda G. Assaraf

**Affiliations:** ^1^ The Fred Wyszkowski Cancer Research Laboratory Department of Biology Technion‐Israel Institute of Technology Haifa Israel; ^2^ Bioinformatics Knowledge Unit The Lorry I. Lokey Interdisciplinary Center for Life Sciences and Engineering Technion‐Israel Institute of Technology Haifa Israel

**Keywords:** breast milk, exclusive breastfeeding, genetic disease, infant, zinc deficiency, zinc transporter

## Abstract

Loss of function (LoF) mutations in the zinc transporter SLC30A2/ZnT2 result in impaired zinc secretion into breast milk consequently causing transient neonatal zinc deficiency (TNZD) in exclusively breastfed infants. However, the frequency of TNZD causing alleles in the general population is yet unknown. Herein, we investigated 115 missense SLC30A2/ZnT2 mutations from the ExAC database, equally distributed in the entire coding region, harboured in 668 alleles in 60 706 healthy individuals of diverse ethnicity. To estimate the frequency of LoF SLC30A2/ZnT2 mutations in the general population, we used bioinformatics tools to predict the potential impact of these mutations on ZnT2 functionality, and corroborated these predictions by a zinc transport assay in human MCF‐7 cells. We found 14 missense mutations that were markedly deleterious to zinc transport. Together with two conspicuous LoF mutations in the ExAC database, 26 SLC30A2/ZnT2 alleles harboured deleterious mutations, suggesting that at least 1 in 2334 newborn infants are at risk to develop TNZD. This high frequency of TNZD mutations combined with the World Health Organization‐promoted increase in the rate of exclusive breastfeeding highlights the importance of genetic screening for inactivating SLC30A2/ZnT2 mutations in the general population for the early diagnosis and prevention of TNZD.

## 1 INTRODUCTION

Zinc is vital for the structure and function of ~10% of the human proteome. As zinc is bound to myriad proteins and sequestered in organelles, the cytoplasmic zinc concentration is very low being at the nmol/L‐pmol/L range.^1^, ^2^ Zinc homoeostasis is tightly regulated by two families of transporter proteins including ZIPs and ZnTs.^2^ ZIPs import zinc into the cytosol from the lumen of organelles or from the extracellular milieu through the plasma membrane.^2^ In contrast, ZnTs compartmentalize zinc within organelles or export zinc to the extracellular milieu. In addition, there are also non‐specific metal chelators that reside in the cytoplasm, termed metallothioneins which efficiently bind zinc.^3^

Tight regulation of the intracellular zinc level is crucial for cell survival and hence for human health. Impaired zinc homoeostasis has an adverse effect on the physiology of the organism, and loss of function (LoF) mutations in zinc transporters lead to various diseases.^4^, ^5^ In this respect, transient neonatal zinc deficiency (TNZD) occurs due to LoF mutations in the SLC30A2 gene encoding for ZnT2.^1^ During lactation, ZnT2 is upregulated in mammary epithelial tissues^6^ where it sequesters zinc within intracellular vesicles.^7^ These secretory vesicles were suggested to fuse to the plasma membrane and exocytose zinc to the milk.^1^, ^8^ Mothers harbouring LoF mutations in SLC30A2/ZnT2, secrete very low levels of zinc into breast milk, leading to zinc deficiency (ie TNZD) in their exclusively breastfed infants.^9^, ^10^, ^11^, ^12^, ^13^, ^14^, ^15^, ^16^ TNZD manifests in infants as severe dermatitis, diarrhoea, alopecia and loss of appetite.^1^ Without zinc supplementation, TNZD can lead to severe anaemia, growth retardation, hypogonadism, skin abnormalities and mental lethargy which can be life‐threatening.^17^, ^18^ Zinc supplementation to the nursing mothers does not increase the zinc levels secreted to the breast milk.^19^ Therefore, the sole treatment for TNZD is early diagnosis and zinc supplementation of the nursing infants, together with continued breastfeeding. We recently showed that a haploinsufficiency state occurs in women with heterozygous SLC30A2/ZnT2 mutations,^20^ indicating that a single SLC30A2/ZnT2 allele with an LoF mutation is sufficient to result in TNZD.^1^, ^10^, ^11^, ^12^, ^21^ The World Health Organization postulates that breastfeeding is the best diet for the health of infants. Thus, the prevalence of TNZD is predicted to increase as more mothers decide to exclusively breastfeed their infants. Characterization of LoF SLC30A2/ZnT2 mutations resulting in TNZD will pave the way towards the development of diagnostic tools. In this respect, in a study with 750 breastfeeding Chinese women it was found that 18 women produced breast milk with very low zinc levels, and in addition five polymorphisms were identified in the SLC30A2/ZnT2 gene.^22^ Moreover, Alam et al, sequenced 54 exomes of women from the USA and found that 38% carried non‐synonymous ZnT2 variants and that these variations were related to higher or lower zinc levels in their breast milk.^23^ Furthermore, Itsumura et al, studied 31 single nucleotide polymorphisms (SNPs) in the SLC30A2/ZnT2 gene that were found in the NCBI database. They found that 4 out of these 31 SNPs had significantly low levels of zinc transport, which were similar to ZnT2 mutations that caused TNZD.^21^ However, data about the prevalence of SLC30A2/ZnT2 mutations in the general population of healthy individuals from different ethnicities, both conspicuous LoF mutations (eg premature translation termination, frameshift and impaired splicing) and missense mutations is lacking. Towards this end, we herein turned to the published ExAC database^24^; according to ExAC containing 60 706 exome sequences of healthy humans, four alleles with conspicuous LoF mutations including gain of premature translation stop codon, splice donor and frameshift were found in the SLC30A2/ZnT2 gene. However, the frameshift mutations were filtered out by ExAC Variant Effect Predictor (supplements data of ref. 24). This indicates that in ExAC, conspicuous LoF mutations in SLC30A2/ZnT2 occur at a frequency of 3/60 706 individuals.^25^ In contrast, missense SLC30A2/ZnT2 mutations occur at a much higher frequency of 1/182, ie 668 alleles with missense mutations out of a total of 121 412 sequenced alleles.^25^ Using bioinformatics, structural modelling of ZnT2, computational prediction of the impact of these mutations on transporter functionality, as well as functional validation assays of loss of zinc transport in live cells, we determined the frequency of TNZD‐causing mutations in the general population. Based on these complementary findings we found that at least 1 in 2334 exclusively breastfed infants will be at risk of developing TNZD.

## 2 MATERIALS AND METHODS

### 2.1 Chemicals and reagents

The DNA dye Hoechst 33342 was purchased from Sigma‐Aldrich Israel (Rehovot, Israel). The cell permeant viable fluorescent zinc probe FluoZin‐3‐AM was from Thermo Fisher Scientific (Waltham, MA, USA). Zinc sulphate was obtained from Merck (Rosh‐Ha'ayin, Israel).

### 2.2 Analysis of exome sequence database

The ExAC exome sequence database (http://exac.broadinstitute.org/)^24^, ^25^ of healthy individuals is an excellent objective source for estimation of the frequency of LoF ZnT2 mutations in the general population and TNZD prevalence as mothers harbouring inactivating ZnT2 mutations that cause TNZD, were not found to have any other related disease and therefore are included in this database. According to the protein atlas database (https://www.proteinatlas.org/ENSG00000158014-SLC30A2/tissue), ZnT2 is expressed only in the kidney, thyroid gland, pancreas and placenta at the mRNA level and is not detected at the protein level in any of these tissues. Based on this information and on the cases that were published in the literature in women harbouring inactivating ZnT2 mutations, we considered these women healthy individuals. ZnT2 expression was demonstrated in rat and mouse tissues or cultured cell lines.^26^, ^27^, ^28^, ^29^, ^30^, ^31^, ^32^, ^33^ Regarding the expression of ZnT2 in human cells, Leung et al,^34^ were the first to show that ZnT2 mRNA levels were readily detected in human retinal ARPE19 cells and in primary foetal RPE cells but not in adult retinal pigment epithelial cells. We have previously shown the expression of ZnT2 in cells freshly isolated from human breast milk samples (Golan et al.^20^). Moreover, Foresta et al,^35^ reported that ZnT2 is expressed in human epididymis epithelial cells. Taking into consideration that the *lethal milk* syndrome in mice is caused by LoF mutations in ZnT4 resulting in similar symptoms to TNZD in humans (caused by inactivating ZnT2 mutations), one can suggest a distinct pattern of ZnT2 expression and/or function in humans and rodents (including mice and rats). Therefore, one cannot assume that ZnT2 expression in a given mouse tissue will be necessarily identical in the cognate human tissue. However, it is possible that in the future, other disease(s) or symptoms will be associated with inactivating ZnT2 mutations, apart from TNZD.

### 2.3 Hypothesis testing

To test the hypothesis that zinc transport is impaired in cells transfected with mutant ZnT2, we compared vesicular FluoZin‐3 fluorescence levels in each of 29 mutants to that of the WT‐ZnT2, using one‐tailed *t* test with unequal variance. Hypothesis testing was followed by False Discovery Rate correction for multiple hypotheses testing with α = 0.05.^36^ To test the hypothesis that the number of FluoZin‐3 vesicles per cell is lower in cells transfected with LoF mutant ZnT2 as compared to cells transfected with the WT‐ZnT2, we compared the number of FluoZin‐3 positive vesicles in each of 11 mutants to the WT‐ZnT2, using one‐tailed *t* test with unequal variance. To test the hypothesis that ZnT2 protein expression is altered in cells transfected with mutant ZnT2 as compared to cells transfected with WT‐ZnT2, we compared Ruby fluorescence levels (actual flow cytometry data and not calculated percentage values) in each of the 29 mutants to the WT‐ZnT2, using two‐tailed paired *t* test. Hypothesis testing was followed by False Discovery Rate correction for multiple hypotheses testing with α = 0.05.^36^

### 2.4 Bioinformatics analysis

Amino acid conservation analysis was performed using the ConSurf tool, which generated an amino acid conservation map of the ZnT2 ORF. ConSurf assigned a conservation range value from 1 to 9 to each amino acid position in ZnT2, based on homologous sequence analysis.^37^, ^38^, ^39^, ^40^ Amino acid positions with a conservation score of 1‐6 were considered “not conserved,” while those with a score of 7 were “somewhat conserved,” score 8 were “conserved,” whereas those with score 9 were considered “very conserved.” One hundred and fifteen SLC30A2/ZnT2 missense mutations from the ExAC database that result in 113 amino acid substitutions were studied using the ConSurf conservation score.

The PROVEAN and Polyphen‐2 tools were utilized to predict whether the missense ZnT2 mutations found in the ExAC database were functionally deleterious. PROVEAN predicts an impact score, calculated based on sequence variation alignment clustering. A mutation with a score less than the cut‐off of −2.5 was considered deleterious to the function of the protein.^41^, ^42^ Whereas PolyPhen‐2 calculates a score for an impact of a mutation on protein function based on homologous sequence clustering algorithm.^43^ The algorithm takes into consideration the conservation of the mutated amino acid, as well as amino acid features like surface area, hydrophobicity, amino acid volume and Ramachandran angles. Polyphen‐2 defines a “possibly damaging” mutation in a score range of 0.45‐0.95, and “probably damaging” in a score range of 0.95‐1.00, while benign mutations are below a score of 0.453.^44^

### 2.5 The thermal stability meta‐predictor tool

The ZnT2 monomer model was aligned to the 3h90 crystal structures template of *YiiP* from *Escherichia coli* (PDB 3h90, chains A and C) by the HHpred method as previously described.^10^ The 3h90 PDB file contains only amino acid residues 73‐277 of human ZnT2; therefore, for the thermal stability evaluation, we studied only the mutations that were contained within this region. The thermal stability meta‐predictor tool was used to predict the effect of missense ZnT2 mutations on the thermal stability of the protein. This tool combines the predictive power of 11 tools to generate two predictive scores, an average from all the tools, as well as a weighted average which takes into consideration the amino acid environment.^45^ The weighted average is considered more accurate.^45^ A score of <−0.2 kcal/mol was considered destabilizing. It is important to note that this tool was trained to predict data on globular proteins and has limited experience with membrane proteins.

### 2.6 Construction of expression vectors

A pcDNA3.1 Zeo (+) expression plasmid encoding for a WT‐ZnT2 tagged with a red fluorescent Ruby protein was generated as described previously.^20^ The mutations were introduced into the ZnT2‐Ruby expression vector using *Pfu* Turbo DNA polymerase (QuikChange kit; Stratagene, La Jolla, CA, USA) and the primers are listed in Table S1.

### 2.7 Cell culture, transient transfections

Human MCF‐7 breast cancer cells were grown and transiently transfected as previously described.^12^, ^46^ FluoZin‐3‐AM, a specific zinc indicator, fluorescently labels zinc‐containing vesicles that were detected solely in cells overexpressing an active ZnT2 transporter. In contrast, cells transfected with an empty RFP vector or non‐active ZnT2 mutants, showed very low levels of zinc accumulation that reflected the low number of FluoZin‐3 positive vesicles per cell. It should be noted that cytosolic zinc levels are very low, and are therefore undetectable using this fluorescent FluoZin‐3 labelling assay.

For the zinc transport experiments, 18 hours after transfection, cells were incubated for 1 hour in growth medium containing 75 μmol/L ZnSO_4_. Cells were then rinsed with PBS and incubated in growth medium containing 1 μmol/L FluoZin‐3‐AM for 45 minutes, following which they were washed twice with PBS and analysed by a Zeiss LSM‐710 (Thornwood, NY, USA) confocal microscope or trypsinized and collected for analysis using a BD FACS Aria IIIu (San Jose, CA, USA) flow cytometer.

### 2.8 Flow cytometric analysis

The mean transfection efficiency was 26% ± 6% and was determined as the percentage of live single cells (after gating for FSC and SSC parameters), displaying Ruby fluorescence levels higher than the untransfected cells (Figure S1). Only cells that showed high levels of Ruby fluorescence were considered as positive for transfection and were analysed for FluoZin‐3 levels. Figure S1 shows the gating parameters that were used for flow cytometry data analysis and a representative dot‐plot of the WT and mutants ZnT2 proteins for Ruby (red fluorescence) vs FluoZin‐3 levels (green fluorescence). At least three independent experiments were performed for each mutant, and 10 000 cells were analysed in each experiment.

### 2.9 Confocal laser microscopy

A magnification of ×63 under immersion oil was used. Excitation wavelengths were 405 nm for Hoechst nuclear DNA labelling, 488 nm for FluoZin‐3, and 543 nm for RFP or Ruby‐tagged ZnT2 proteins.

### 2.10 Imaris analysis for FluoZin‐3 vesicles co‐localizing with ZnT2‐Ruby proteins

We used the Imaris software version 8.41 spots module with basic Matlab script for co‐localization of fluorescent punctate structures. The threshold for the detection of both ZnT2‐Ruby and FluoZin‐3 punctate structures was set using the WT‐ZnT2 confocal image. We next calculated the number of the FluoZin‐3 dots that co‐localized with the ZnT2‐Ruby dots per cell, for the WT‐ZnT2 images and compared it to the other LoF ZnT2 mutants images. This number of co‐localized vesicles per cell is a direct reflection of the zinc accumulation capacity via the Ruby tagged‐ZnT2 transporter. At least five different confocal images were analysed for each mutant with at least 15 cells.

## 3 RESULTS

### 3.1 Bioinformatics analysis of missense ZnT2 mutations predicts that 45 mutations have a deleterious impact on transporter stability and transport function

In order to determine the frequency of LoF missense SLC30A2/ZnT2 mutations that are causative of TNZD in the general population, we analysed the ExAC database. According to this database, in 60 706 healthy humans, four conspicuous LoF alleles including gain of premature translation stop codon, splice donor and frameshift mutations were found in the SLC30A2/ZnT2 gene. Only three of these alleles were considered LoF after filtering out false‐positive variants, and the frameshift mutation was filtered out by ExAC Variant Effect Predictor (supplementary data of reference 24). Therefore, conspicuous LoF mutations in SLC30A2/ZnT2 are rare as their frequency according to the ExAC database is 3/60 706 individuals.^25^ Expectedly, missense SLC30A2/ZnT2 mutations occur at a much higher frequency of 1/182 (668 in 121 412 alleles harbouring 115 mutations). For consistent estimation of risk allele frequency in the general population, we used the maximal number of sequenced alleles which is 121 412. On average, 119 997 alleles were sequenced; hence the deleterious allele frequency in the population may be higher than our conservative estimate.

**Figure 1:**
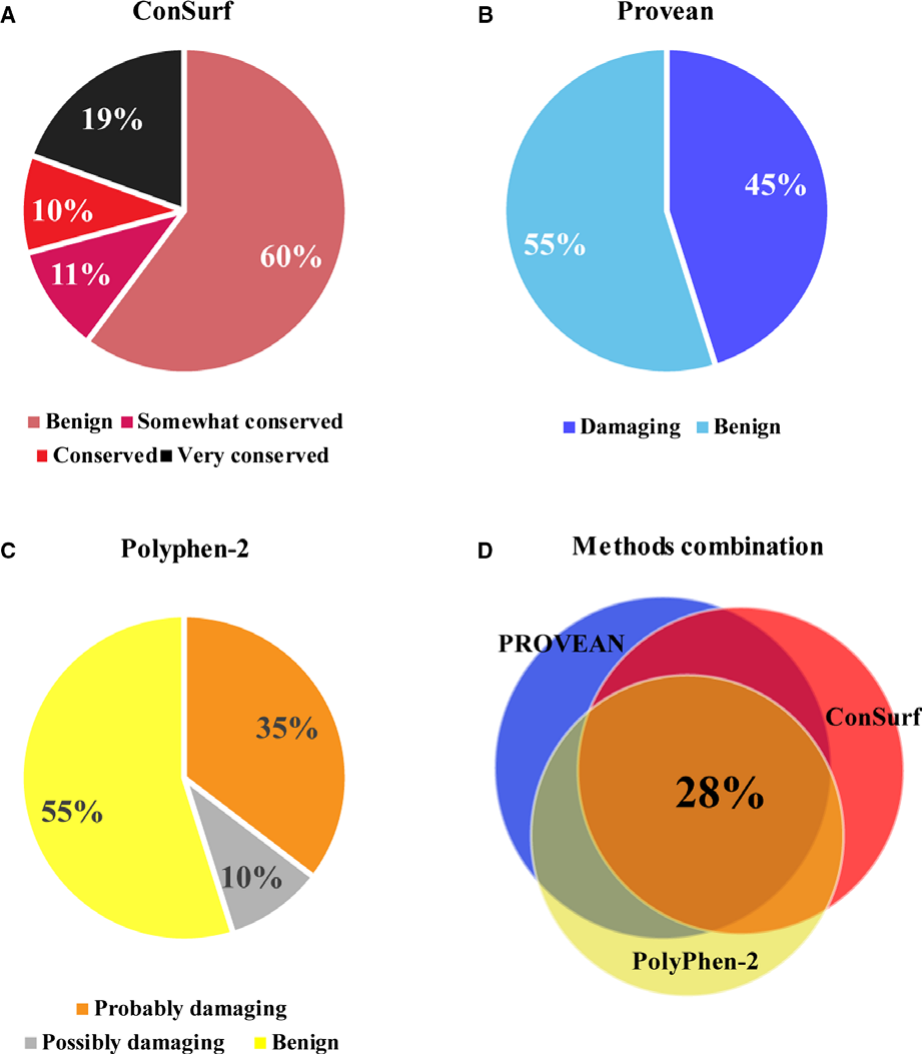
Conservation prediction of amino acid residues mutated in ZnT2 and the predicted effect of these ZnT2 mutations on zinc transport function. A, Conservation rates of 113 missense mutations that were found in ZnT2 in the ExAC database and were analysed using ConSurf software. PROVEAN and PolyPhen‐2 prediction of 113 ZnT2 missense mutations which appear to have a deleterious effect (B and C, respectively). D, Venn diagram showing the percentage of mutations in conserved residues (based on ConSurf analysis) that were predicted to have a deleterious effect on ZnT2 function using PROVEAN and PolyPhen‐2 analyses

To evaluate the impact of these missense mutations on ZnT2 function, we first analysed the conservation of the different residues that were substituted, as substitution of conserved residues markedly increases their probability to be deleterious to function. The 115 nucleotide mutations lead to 113 amino acid substitutions. According to ConSurf, 40% of the missense mutations listed in the ExAC database occur in conserved regions, with 19% of all mutations mapping to highly conserved residues (Figure 1A). We further used a computational method to predict whether the mutations were deleterious. Using PROVEAN analysis and PolyPhen‐2 data, we found that 45% of the 113 ZnT2 missense mutations were predicted to be deleterious to function (Figure 1B and C). To narrow down the list of ZnT2 missense mutations considered to be deleterious, we cross‐analysed the computational prediction data of ConSurf, PROVEAN, and PolyPhen‐2 for all the 113 ZnT2 missense mutations. Thirty‐two missense ZnT2 mutations, ie 28% out of the 113 mutations studied, were predicted to be deleterious by both PROVEAN and PolyPhen‐2, as well as by ConSurf (Figure 1D). Forty‐five mutations, or 39% of the 113 mutations, were predicted to be deleterious by both PROVEAN and PolyPhen‐2 analyses; these mutations are presented in Table 1. In order to select ZnT2 missense mutations to be assayed for actual zinc transport capacity, thermal stability meta‐predictions, structural analysis and literature comparison were performed on the mutations listed in Table 1.

**Table 1:** Degree of conservation and thermal stability of 45 mutations that were predicted to be deleterious for ZnT2 function based on PROVEAN and PolyPhen2 analyses. The # symbol near the allele number represents mutations that were assayed in the zinc transport assay, whereas mutations that showed impaired zinc transport are marked with an asterisk

	Deleterious ZnT2 mutations predicted by PROVEAN and PolyPhen‐2	Consurf	Simple average (kcal/mol)	Meta prediction (kcal/mol)	Allele count	Explanation for choosing this residue for examination
1	Y19S				1	
2	H54Y	Very conserved			1#	H54R mutation was previously reported to cause TNZD^11^
3	R72C				3#	Based on 3D model located near the entrance to the zinc permeation pathway
4	R72H				3#	
5	M85I	Very conserved	−0.17	0.95	2#	Conservation
6	G87R		−0.14	−0.04	5*	G87R mutation was previously reported to cause TNZD^9^, ^16^
7	T102A	Conserved	−0.68	−0.2	1	
8	A104S	Very conserved	−0.32	−0.07	2#	Thermal stability
9	A104T	Very conserved	0.47	0.65	2	
10	H106Y	Very conserved	0.22	0.82	2*	H106 is part of the predicted zinc binding site in ZnT2 (parallel to site A in YiiP^61^)
11	S113T	Conserved	0	0.91	1	
12	W122C		−2.89	−3.18	5#	Thermal stability
13	R126Q	Somewhat conserved	−0.57	−0.48	3	
14	A144S	Very conserved	−1.06	−1.18	2#	Thermal stability
15	V154M	Conserved	−0.04	0.52	3	
16	G156V	Very conserved	0.66	0.65	1#	Conservation
17	R165W	Very conserved	0.91	1.69	1*	Conservation
18	R165Q	Very conserved	−0.25	0.39	1	
19	G175R		1.78	1.7	12#	Major substitution of this residue
20	G175W		0.96	0.85	3*	Major substitution of this residue
21	T181M	Very conserved	0.67	1.35	1	
22	A185T	Conserved	−1.41	−0.22	3	
23	N189K	Very conserved	0.21	0.98	1	
24	I190T		−2.45	−1.94	1#	Thermal stability
25	H197R	Conserved	0.11	0.88	1	
26	H205D	Very conserved	0.29	0.22	1	
27	N214K	Very conserved	−0.17	0.4	1*	Conservation
28	R218Q	Very conserved	−0.7	−0.31	4#	Conservation
29	G226S	Very conserved	0.23	0.98	4#	Conservation
30	S231T	Very conserved	−1.14	−0.39	1#	Conservation and thermal stability
31	G233R	Very conserved	−0.2	0.61	1*	Conservation and thermal stability
32	G233D	Very conserved	−0.28	−0.06	1*	Conservation and thermal stability and previous publication^21^
33	P245R	Conserved	−1.31	−1.46	1*	Thermal stability
34	E246K		−0.43	0.41	1#	Thermal stability
35	S259A	Very conserved	1.18	1.83	1	
36	I269T	Conserved	−1.05	−1.35	1#	Thermal stability
37	E279K	Very conserved			1*	Conservation and proximity to G280 that was shown to be important for ZnT2 function^12^
38	R291H				84	
39	G299R	Somewhat conserved			9#	Major change in this residue
40	G299W	Somewhat conserved			3*	Major change in this residue
41	V300L	Very conserved			1*	Conservation
42	A310V	Somewhat conserved			1	
43	A323T	Somewhat conserved			1	
44	V333M	Somewhat conserved			2	
45	E355K	Very conserved			1	E355K and E355Q were previously reported to cause TNZD^12^, ^15^

### 3.2 Eleven missense ZnT2 mutations show a markedly deleterious effect on zinc transport capacity

**Figure 2:**
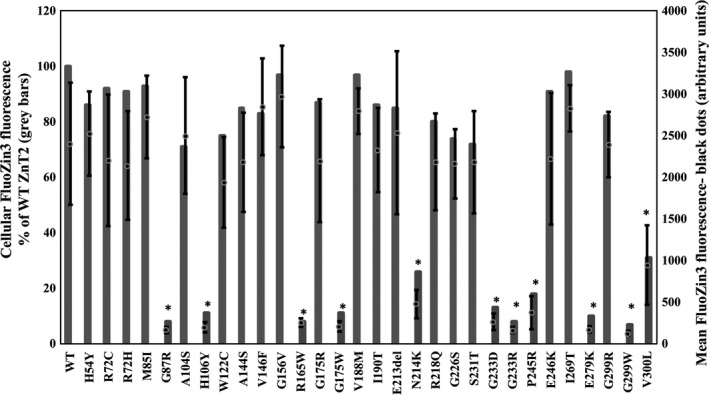
Zinc accumulation capacity of ZnT2 mutants which were found in the ExAC database and were predicted to be deleterious to function. MCF‐7 cells transiently transfected with the ZnT2‐Ruby constructs containing the mutations depicted along the *X* axis, were examined for FluoZin‐3 fluorescence levels, which reflect actual vesicular zinc accumulation. FluoZin‐3 fluorescence was determined using flow cytometry only for cells displaying Ruby tagged‐ZnT2 fluorescence (ie positively transfected cells) and not for the entire cell population. Gray bars represent cellular FluoZin‐3 fluorescence as % of WT‐ZnT2 accumulation. The black dots represent the mean FluoZin‐3 fluorescence levels of the different mutants in transfected cells as determined by flow cytometry. Error bars represent SD of at least three independent experiments. Asterisks indicate that the values obtained are significantly lower than WT‐ZnT2 (*t* test with FDR, α = 0.05)

We explored the ability of 29 ZnT2 mutants to accumulate zinc in intracellular vesicles as indicated by the fluorescence intensity of the specific zinc fluorophore FluoZin‐3 in MCF‐7 cells transiently transfected with expression vectors harbouring these ZnT2 mutations. In this assay, high levels of FluoZin‐3 fluorescence are observed in a high number of intracellular vesicles solely in cells overexpressing active ZnT2 transporter. Eleven out of 29 mutants that were tested failed to accumulate zinc and displayed only residual zinc accumulation of 10%‐25% compared to the WT‐ZnT2 protein as determined by flow cytometry (Figure 2). The G87R mutant found in the ExAC database served as a *bona fide* LoF control as it was previously shown to cause TNZD.^16^, ^46^ In addition to the 29 missense mutations that we tested for zinc function, we also studied the in‐frame deletion of three nucleotides which resulted in deletion of residue E213 in ZnT2. Remarkably, this in‐frame deletion was found in 17 alleles in the ExAC database. Because of the high frequency of this deletion we assessed whether or not it has a deleterious effect on ZnT2 function. E213del mutant expression and its ability to transport zinc into intracellular vesicles were similar to those of the WT‐ZnT2 (Figure 2), indicating that this variant retains its normal zinc transport function. Based on the haploinsufficiency state that occurs in women with heterozygous SLC30A2/ZnT2 mutations,^20^ these missense LoF ZnT2 mutations are predicted to be sufficient to markedly decrease zinc concentration in breast milk, thereby causing TNZD in exclusively breastfed infants.^1^

### 3.3 Three out of the 11 LoF mutations had low expression levels compared to the WT protein as indicated by low Ruby tagged‐ZnT2 fluorescence levels

**Figure 3:**
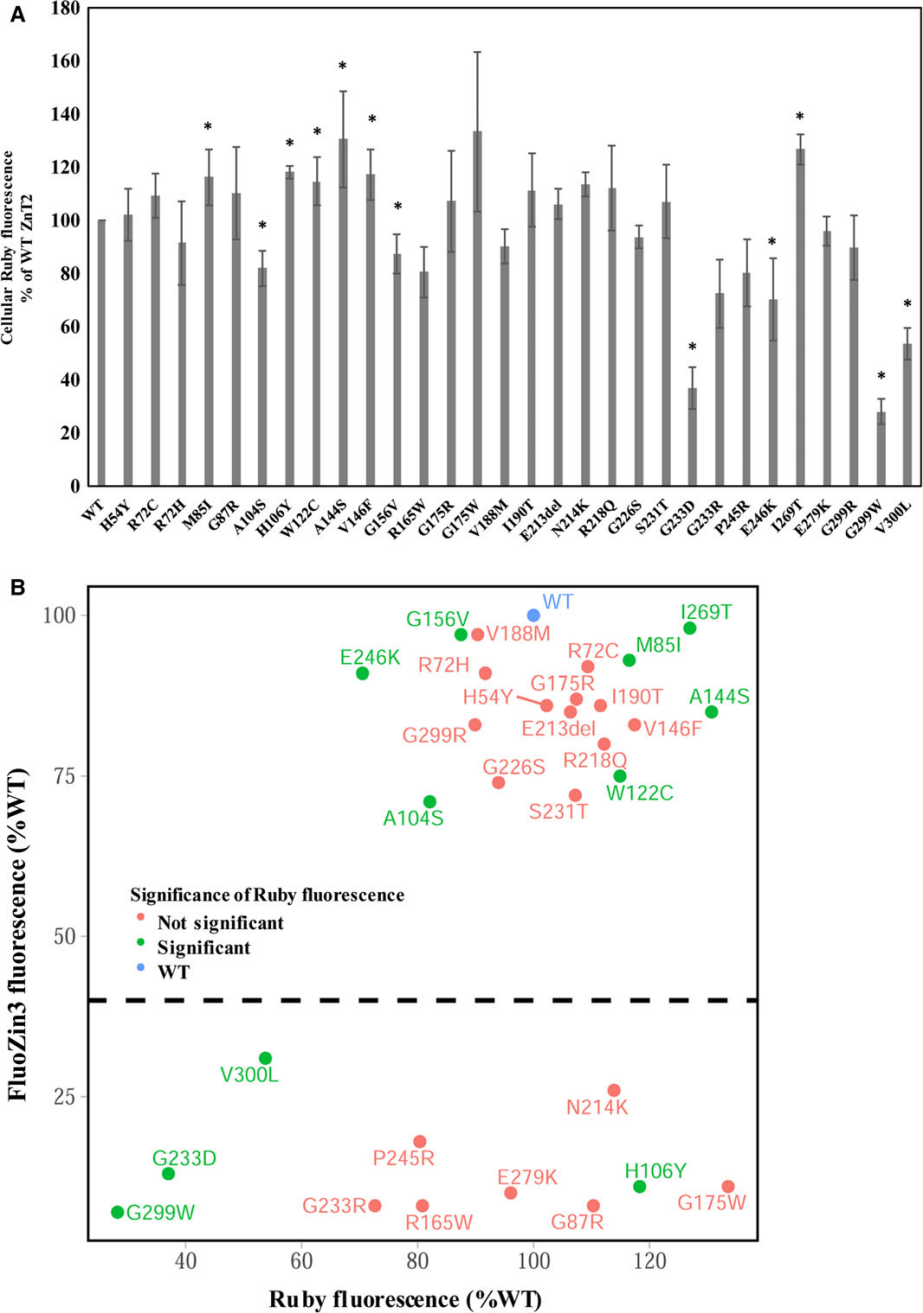
G233D, G299W and V300L ZnT2 mutants exhibit decreased protein expression or increased degradation. A, MCF‐7 cells transiently transfected with the ZnT2‐Ruby constructs containing the mutations depicted along the *X* axis, were examined for Ruby fluorescence levels. Ruby fluorescence was determined using flow cytometry only for cells displaying Ruby tagged‐ZnT2 fluorescence above the level of none‐transfected cells (ie positively transfected cells) and not for the entire cell population. Bars represent the fluorescence as % of the WT‐ZnT2 fluorescence. Error bars represent SD of at least three independent experiments. Asterisks indicate that the values obtained are significantly lower or higher than WT‐ZnT2 (*t* test with FDR, α = 0.05). B, Scatter plot of FluoZin‐3 (*Y* axis) versus Ruby fluorescence (*X* axis) as % of WT‐ZnT2 of the various mutants that were functionally examined. All the mutants appearing below the dashed line had FluoZin‐3 values that were significantly lower when compared to the WT‐ZnT2 (*t* test with FDR, α = 0.05). Mutants that were stained in red colour were not significantly different from the WT in their Ruby fluorescence values, whereas mutants coloured in green had Ruby fluorescence levels lower or higher, when compared to the WT‐ZnT2 levels. Evidently, Ruby fluorescence levels vary between mutants with significantly lower FluoZin‐3 levels. Therefore, impaired transport is not necessarily due to change in ZnT2 expression

We next analysed the expression levels of these 29 mutants in order to determine whether low protein expression levels or increased degradation rates, underlie the markedly decreased zinc transport function. Towards this end, cellular Ruby‐tagged ZnT2 fluorescence levels were determined by flow cytometry after transfection with expression plasmids harbouring the different mutants. We found that three out of the mutants which showed low levels of FluoZin‐3 accumulation (G233D, G299W, and V300L) had Ruby fluorescence levels that were significantly lower when compared to the WT‐ZnT2 (Figure 3A). In contrast, six different mutants (M85I, H106Y, W122C, A144C, V146 and I269T) displayed significantly higher levels of cellular Ruby fluorescence, when compared to the WT‐ZnT2. Scatter plot of FluoZin‐3 versus Ruby fluorescence in the different mutants (Figure 3B) clearly indicates that the impaired zinc transport function of the different LoF mutants (in the lower part of the dot‐plot in Figure 3B) is not necessarily a result of a low expression or increased ZnT2 degradation, as only three mutants display decreased Ruby levels. The remaining eight mutants with low FluoZin‐3 fluorescence levels, displayed similar or higher (H106Y) protein levels when compared to the WT‐ZnT2. These mutants probably lost their function either due to alterations in the zinc binding, zinc permeation and/or loss of dimerization.

### 3.4 Five LoF mutants lost their canonical vesicular localization

**Figure 4:**
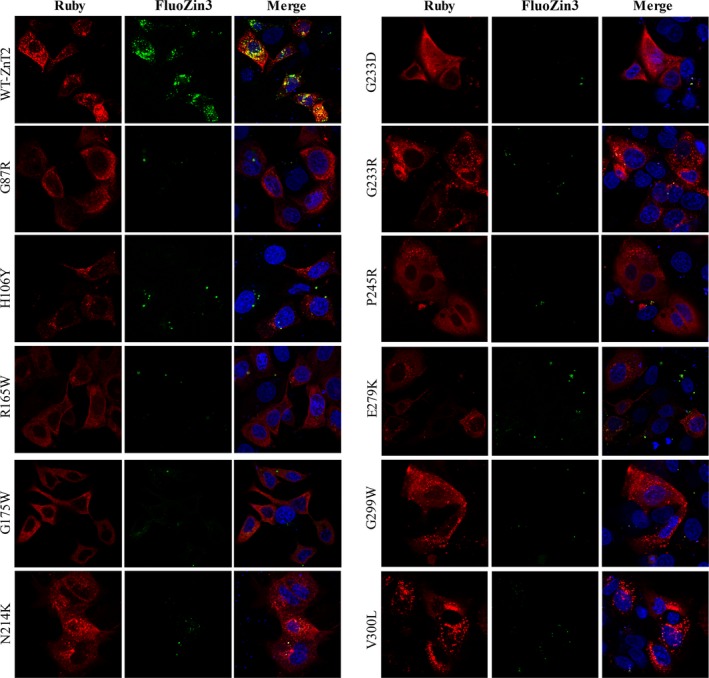
Intracellular localization of the functionally verified ExAC LoF ZnT2 mutants. FluoZin‐3 (green fluorescence) indicates vesicular zinc accumulation. Red fluorescence represents the WT‐ZnT2‐Ruby, or the different Ruby‐tagged mutant ZnT2 proteins as indicated in the left legends. Hoechst 33342 (blue fluorescence) was used to label nuclei. A magnification of ×63 under immersion oil was used. For detection of the G233D, G233R, P245R, G299W and V300L mutants, we used a higher 543 nm laser excitation. The 488 nm laser remained constant in all the photographs in order to be able to properly compare FluoZin‐3 levels

**Figure 5:**
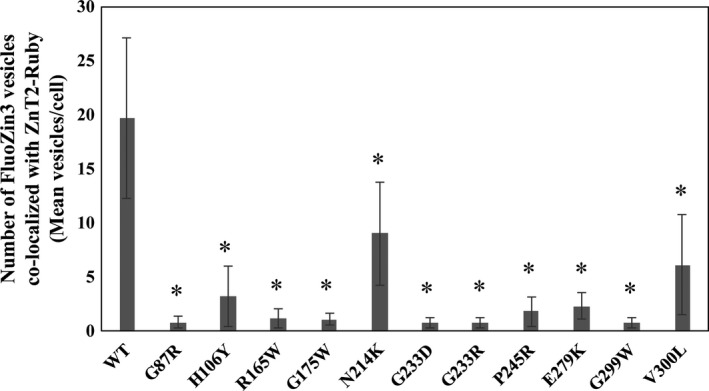
**impaired vesicular zinc accumulation of the functionally verified ExAC LoF ZnT2 mutants**. Imaris software spots module with basic Matlab script for spots co‐localization was used for vesicular co‐localization analysis. The number of the FluoZin‐3 dots that co‐localized with the ZnT2‐Ruby dots was calculated for the WT‐ZnT2 and the other LoF ZnT2 mutants. The number of co‐localized vesicles per cell is a direct reflection of the zinc accumulation capacity via the Ruby tagged‐ZnT2 transporter. Asterisks indicate that the values obtained are significantly lower than WT‐ZnT2 (*p*<0.05).

We further studied the subcellular localization of the 11 mutants which displayed very low levels of zinc accumulation using confocal microscopy. Five of these mutants (G233D, G233R, P245R, G299W and V300L) exhibited low levels of Ruby‐tagged ZnT2 fluorescence (thus, in order to detect them we used higher laser excitation intensity) (Figure 4). In addition, R165W, G175W, G233D, P245R and E279K failed to reach their canonical vesicular localization as was shown here and previously for the G87R ZnT2 mutant (Figure 4).^46^ As is the case for this G87R mutant, we propose that mutants displaying an impaired localization phenotype could possibly exert a dominant negative effect over the WT‐ZnT2 upon homodimerization.^46^ In order to provide statistical confirmation regarding the confocal microscopy data, we used the Imaris software for vesicular co‐localization analysis. We set a threshold for the detection of both ZnT2‐Ruby vesicles and FluoZin‐3 vesicles using the WT‐ZnT2 image. We next calculated the number of the FluoZin‐3 vesicles that co‐localized with the ZnT2‐Ruby vesicles for the WT‐ZnT2 and compared it to the other LoF ZnT2 mutants. This number of co‐localized vesicles per cell is a direct reflection of the zinc accumulation capacity via the Ruby tagged‐ZnT2 transporter. All the mutants that were found to be inactive in zinc transport displayed a significantly decreased number of co‐localized vesicles per cell when compared to the WT‐ZnT2 (Figure 5).

### 3.5 The frequency of LoF ZnT2 mutations which can lead to TNZD in the general population is higher than 1/2334

To estimate a minimal frequency of SLC30A2/ZnT2 alleles harbouring deleterious missense mutations in the general population of healthy individuals, we combined the frequency of LoF mutations from the ExAC database with the frequency of missense mutations that we functionally verified here to be inactive in zinc transport. We also considered the p.E355K mutant that was found in the ExAC database (Table 1) as an inactivating mutation as this heterozygous mutation was reported to cause TNZD in an exclusively breastfed infant.^15^ Two additional mutants that were predicted to have a deleterious effect based on our bioinformatics analysis, were previously shown to be inactive in zinc transport, to the same extent as ZnT2 mutants that cause TNZD.^21^ These two mutations, T181M and N189K, were added to the deleterious missense ZnT2 mutations in our analysis.^21^ Out of 121 412 alleles that were sequenced, 668 alleles harboured 113 unique missense mutations. Out of these 113 mutations, 45 (39%) that were predicted to be deleterious to ZnT2 function, were harboured in 181 alleles (27%). As abovementioned, 29 out of the 45 alleles were functionally tested in a zinc transport assay, 11 of which showed a marked deleterious impact with ≥75% reduction in zinc transport activity. Three mutations that were not tested were previously reported as causal for TNZD (p.E355K)^15^ or low zinc transport (T181M and N189K^21^). In total, 26 alleles harboured the 14 highly deleterious missense ZnT2 mutations along with two conspicuous LoF mutations (premature stop codon and splice donor). Notably, there were no homozygous mutations in ZnT2 in the ExAC database. In addition, ZnT2 mutations tend to be dominant due to haploinsufficiency of the WT allele that we and others have previously shown.^1^, ^10^, ^11^, ^12^, ^20^, ^21^ Based on these composite findings, the rate of risk allele carriers is estimated at 26/60 706 individuals or 1/2334 individuals. This frequency is an underestimation as it is expected that more deleterious mutations are present within the 14 mutations that were predicted to be deleterious but were not functionally assessed. Moreover, there may be more deleterious alleles within the 68 mutations that were not predicted to be deleterious, due to false negative errors of the prediction algorithms.

## 4 DISCUSSION

In this study, we aimed at determining the frequency of TNZD‐causing mutations in the general population. An assortment of TNZD cases was reported in the past 12 years,^1^ as the first LoF ZnT2 mutation was found to be causative of TNZD^11^. Therefore, we previously emphasized the importance of the early diagnosis of TNZD in order to prevent mild and severe zinc deficiency in infants.^1^, ^20^ To date, cases of TNZD are diagnosed only after the symptoms of severe zinc deficiency appear in the infants. However, we assume that many cases of mild zinc deficiency can be masked when the babies are frequently consuming zinc‐containing formulas and supplementary foods,^1^, ^47^ thereby remaining completely undiagnosed. Therefore, it is important to develop a reliable, rapid, and relatively facile genetic test for the early diagnosis of TNZD, especially due to the fact that the treatment is simple and requires only zinc supplementation of the infant's diet. Upon identification of a LoF ZnT2 mutation, a mother should supplement her breastfed infants with zinc upon the first and next deliveries and hence there is no need for further genetic testing. The other alternative for early diagnosis, is to determine zinc levels in the breast milk; however, the large inter‐individual variability in zinc concentration in breast milk and the decreasing zinc levels along lactation,^48^ along with the fact that there is no routine test for the reliable quantification of zinc concentration in breast milk, render this quantitative tool highly inadequate. Other genetic and metabolic diseases are routinely tested in newborn infants, although their frequencies are much lower compared to the prevalence that we report herein for TNZD mutations. A few examples include screening tests which are performed using blood drop from the newborn infant for the detection of phenylketonuria which occurs with a prevalence of 1 in 10 000‐15 000 newborns in the USA,^49^ maple syrup urine disease which is estimated to occur with a prevalence of 1 in 185 000 infants worldwide,^50^, ^51^ and propionic acidemia which affects ~1 in 100 000 individuals in the USA.^52^, ^53^, ^54^

**Figure 6:**
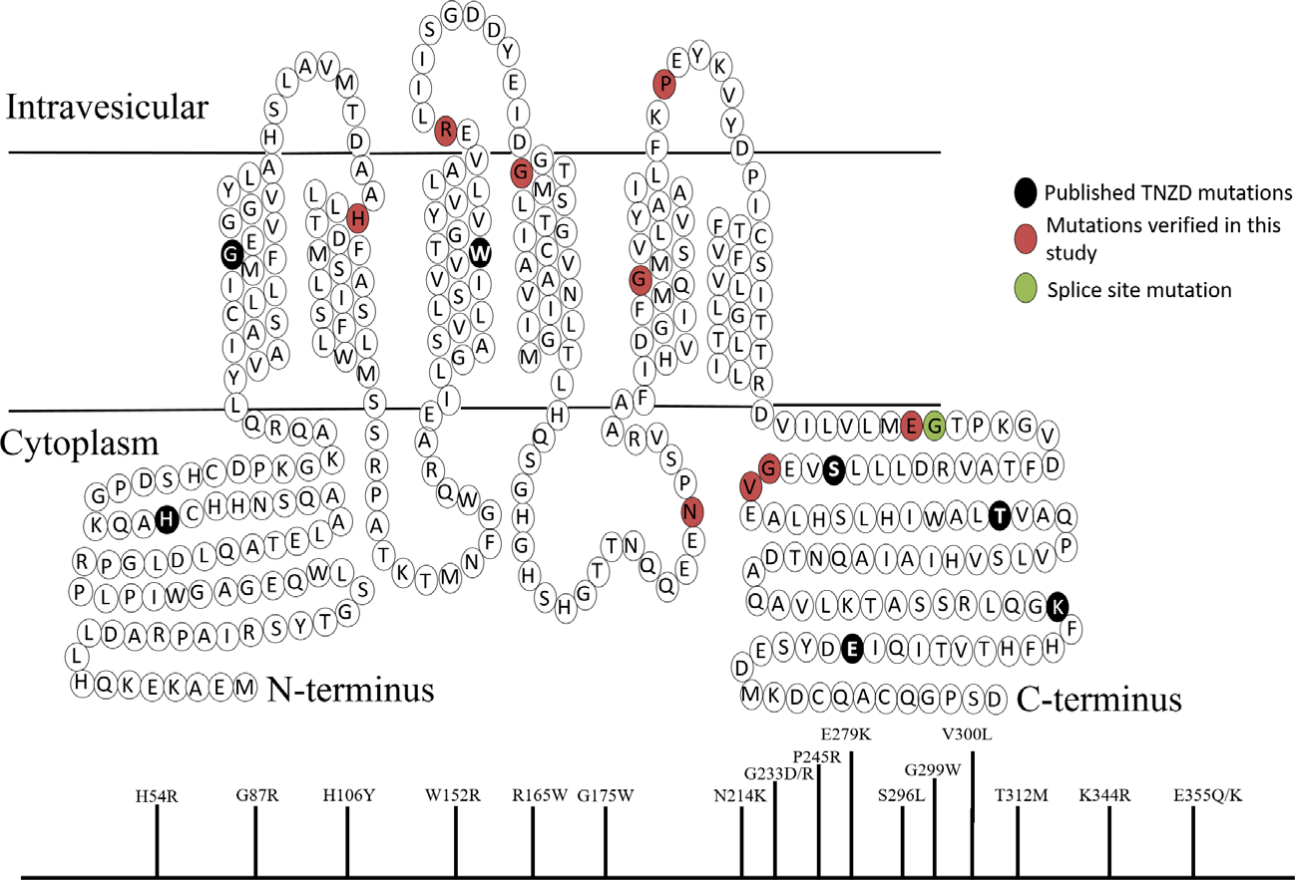
Schematic topology of ZnT2 and the localization of all known LoF mutations along the ZnT2 transporter

The ExAC database that we used herein is ideal for estimating the prevalence of TNZD risk, as mothers harbouring inactivating ZnT2 mutations which are not associated with any other related disorder, were included in this database which represents exome sequences of healthy individuals of diverse ethnicity. However, it is possible that inactivating ZnT2 mutations cause other rare disease(s) that are yet uncharacterized. Nevertheless, the primary aim of our current study was to estimate the frequency of individuals in the general and healthy population that might be at risk for producing zinc deficient breast milk. Taking into consideration the role of ZnT2 in the development and involution process of the mouse mammary gland,^55^, ^56^ it is also conceivable that LoF ZnT2 mutations in humans will influence other important processes in the mammary gland, that will have other implications apart from TNZD in these healthy individuals. Although distinct ethnic groups are represented in the ExAC study, it is not exhaustive and other genetic variations exist in the population. For example, at least eight mutations that were reported in the literature to cause TNZD were not found in the ExAC database.^10^, ^11^, ^12^, ^14^, ^20^, ^21^, ^57^ Therefore, the prevalence of TNZD risk in the worldwide population is likely to be higher than our current estimation, implying that a higher number of infants are at a significant risk for zinc deficiency in the first and critical months of life which can affect their growth, development and immune system function. However, it is important to note that the representation of each inactivating TNZD mutation is certainly not frequent. Herein we assumed that the overall rate of deleterious ZnT2 mutations that we identified is lower bound estimation for the occurrence of deleterious ZnT2 alleles in the population. In addition to mutations that appear only once in the ExAC database, some inactivating ZnT2 mutations were found in a higher frequency in the tested population like the G87R mutation which appeared 5 times in the ExAC database. Two mutations were previously reported to result in a G87R substitution,^9^, ^16^ and the G87R mutation was previously shown to have a dominant negative effect over the WT allele, due to the functionality of ZnT2 as a homodimer.^16^, ^46^ Moreover, a SNP in SLC30A2/ZnT2 which results in a L23P substitution was found at a high frequency in the ExAC database, with 136 alleles containing this SNP out of 119 620 alleles that were sequenced, indicating a prevalence of 1 out of 440 individuals carrying this SNP. This SNP was previously shown to cause mis‐localization of ZnT2 into lysosomes^58^ and in an additional study, a Chinese woman harbouring this SNP was found to secrete low levels of zinc into her breast milk.^22^ This SNP was found mainly in Africans (133 out of 136 alleles with this SNP), and if indeed carrying this SNP is indicative of low levels of zinc in mother's breast milk, it may dramatically increase the prevalence of TNZD in this specific ethnic group. To assess if LoF ZnT2 mutations occur in the form of a mutation hotspot known as mutation cluster region,^59^, ^60^ we generated Figure 6, which summarizes all the LoF ZnT2 point mutations that were reported to date, including those identified in the present study. Interestingly, these inactivating mutations were found to affect each domain of the transporter without any apparent mutation cluster region.

The observation that deleterious alleles are accumulated in ZnT2 gains support from the moderate probability of LoF intolerance (pLI) of ZnT2. pLI is calculated for genes of sufficient length, it ranges between 0 and 1, with 0 indicating tolerance to LoF mutations and one indicating intolerance to LoF mutations. pLI distribution is bimodal with 10 374 genes having pLI ≤0.1 and 3230 genes with pLI ≥ 0.9. Among the families of zinc transporters including SLC30 and SLC39, four genes display pLI scores >0.9; these include the ZnTs SLC30A1/ZnT1, SLC30A10/ZnT10, and SLC30A4/ZnT4 as well as the SLC39A10/Zip10 (Figure S2). For SLC30A2/ZnT2, 12.5 LoF mutations were predicted and only two conspicuous LoF mutations were found in 60 706 people, resulting in a pLI of 0.71 (Figure S2)^25^ which indicates moderate intolerance to LoF. This means that there is a selection against inactivating ZnT2 mutations; however, some mutations appear to evade this selection for reasons discussed below. As pLI is modestly correlated with gene length, we further compared the pLI of certain SLCs to the pLI of genes with similar length. To this end, we divided the ExAC database into 10 groups, each containing 10% of the genes, with increasing gene length (Figure S3). SLC30A2/ZnT2 which belongs to the group of ~1194 bp long genes, had a significantly higher pLI score as compared to the average pLI of this group (*P* < 0.001 confidence interval, Figure S3). Therefore, a pLI of 0.71 for SLC30A2/ZnT2 supports a moderate intolerance to LoF mutations, that is higher than most of the SLC30A and SLC39A genes, and is independent of its relatively short length as its pLI score is higher than most of the genes in this gene length group (Figures S2 and S3). The moderate pLI score of SLC30A2/ZnT2 implies that this gene is undergoing a purifying selection (ie selection against inactivating mutations) as compared to other ZnTs, albeit this selection is not as strong as in ZnT1, ZnT4 and ZnT10. At least two possible mechanisms can explain this moderated selection: first, the purifying selection occurs only when the mother carries the mutation and not when the father harbours the mutation. Second, the selection does not occur in the carrier but only in the next generation, ie the offspring in which the disease is manifested.

In summary, our current analysis reveals that a relatively large fraction of individuals in the general population, harbours LoF ZnT2 mutations. We used an unbiased analysis which is based on published, large scale exome sequence database that includes diverse ethnic groups, gender, and solely healthy individuals. Hence, this analysis can predict the minimal frequency of LoF ZnT2 mutations in the general population, and the number of infants at high risk for developing TNZD. These findings highlight the necessity and importance of instigating a genetic screening test in mothers aimed at the early diagnosis of TNZD in the worldwide population, hence providing a real time zinc supplementation that will markedly eliminate the emergence of TNZD cases.

## CONFLICT OF INTEREST

The authors confirm that there are no conflicts of interest.

## AUTHOR CONTRIBUTION

Y.G. and Y.G.A. designed the research; Y.G. and A.L. performed zinc accumulation assays shown in Figures 4 and 5; A.L performed bioinformatics analysis summarized in Figure 1; G.H. analyzed the ExAC database, performed the statistical analysis and prepared Figures S1‐S3; Y.G. and Y.G.A. wrote the paper. All the authors read and approved the final revised version of the submitted paper.
