# Supplementary figures and images for: High proportion of transient neonatal zinc deficiency causing alleles in the general population
Source: J Cell Mol Med. 2018 Nov 18;23(2):828–40. doi: 10.1111/jcmm.13982 (PMC6349188; doi:10.1111/jcmm.13982)

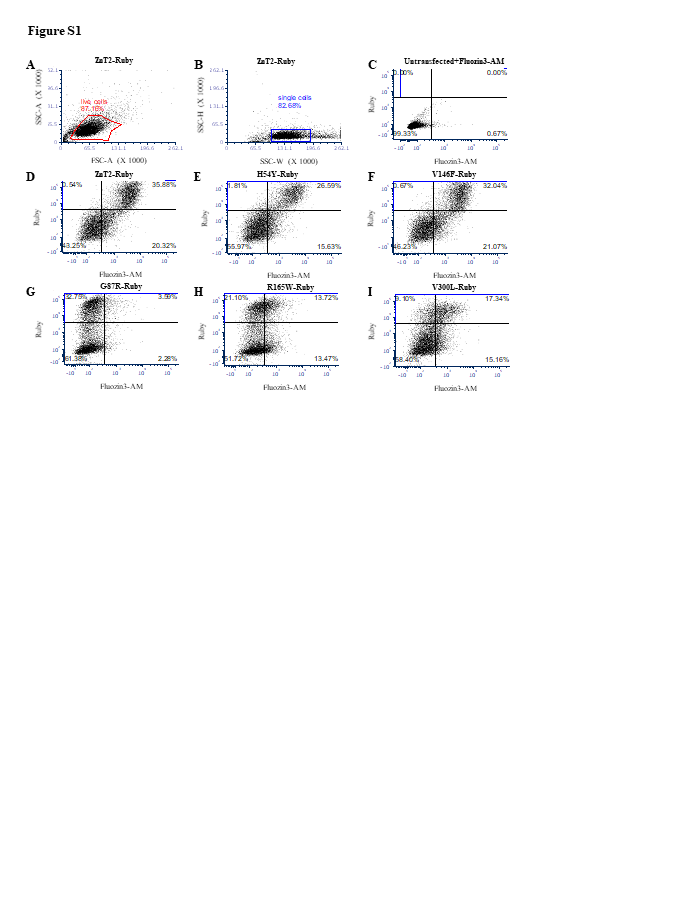

Supplement: Supplementary file 1 [file JCMM-23-828-s001.tif]

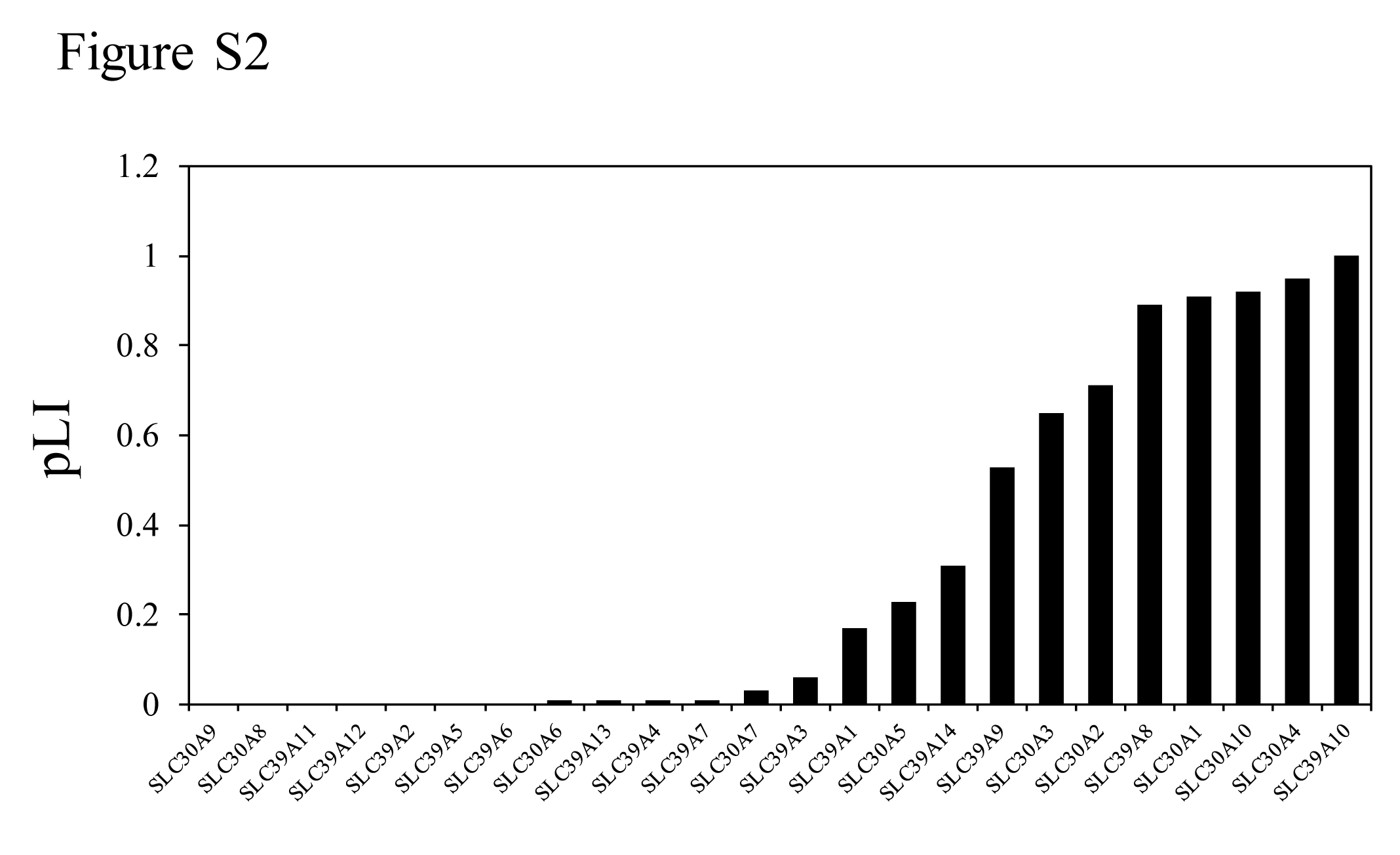

Supplement: Supplementary file 2 [file JCMM-23-828-s002.tif]

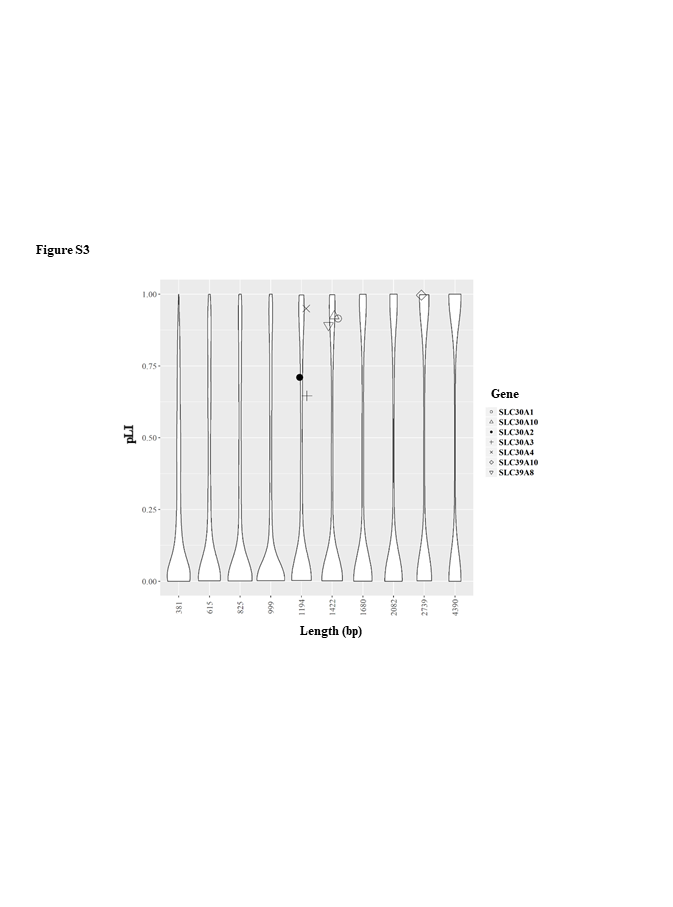

Supplement: Supplementary file 3 [file JCMM-23-828-s003.tif]
