# Supplementary material for: High proportion of transient neonatal zinc deficiency causing alleles in the general population
Source: J Cell Mol Med. 2018 Nov 18;23(2):828–40. doi: 10.1111/jcmm.13982 (PMC6349188; doi:10.1111/jcmm.13982)
Supplement: Supplementary file 4 [file JCMM-23-828-s004.docx]

|  | **Table 1S: Primers for introduce mutations into ZnT2** | | |
| --- | --- | --- | --- |
|  | Name | Forward 5' to 3' | Reverse 5' to 3' |
| 1 | H54Y | AGCAACCATCACTGCTATGCTCAGAAGGGTCCT | AGGACCCTTCTGAGCATAGCAGTGATGGTTGCT |
| 2 | R72C | AAGAAGGGGAAGGCCCAGTGCCAGCTGTATGTAGCC | GGCTACATACAGCTGGCACTGGGCCTTCCCCTTCTT |
| 3 | R72H | GGGAAGGCCCAGCACCAGCTGTATGTAGC | GGCTACATACAGCTGGTGCTGGGCCTTCCC |
| 4 | M85I | GCCATCTGCCTGTTGTTCATCATCGGAGAAGTCG | CGACTTCTCCGATGATGAACAACAGGCAGATGGC |
| 5 | A104S | CTGTCATGACTGACTCAGCACACCTGCTCACT | AGTGAGCAGGTGTGCTGAGTCAGTCATGACAG |
| 6 | H106Y | ATGACTGACGCCGCATACCTGCTCACTGACTTTGCC | GGCAAAGTCAGTGAGCAGGTATGCGGCGTCAGTCAT |
| 7 | W122C | CCTCTTCTCCCTCTGCATGTCCTCCCGG | CCGGGAGGACATGCAGAGGGAGAAGAGG |
| 8 | A144S | CTGAGATCTTGGGATCCCTGGTCTCTGTACTGTC | GACAGTACAGAGACCAGGGATCCCAAGATCTCAG |
| 9 | V146F | CTTGGGAGCCCTGTTCTCTGTACTGTCCATC | GATGGACAGTACAGAGAACAGGGCTCCCAAG |
| 10 | G156V | CTGGGTCGTGACGGTGGTACTGGTGTAC | GTACACCAGTACCACCGTCACGACCCAG |
| 11 | R165W | CTGGCTGTGGAGTGGCTGATCTCTGGG | CCCAGAGATCAGCCACTCCACAGCCAG |
| 12 | G175R | GACTATGAAATTGACCGGGGGACCATGCTGATC | GATCAGCATGGTCCCCCGGTCAATTTCATAGTC |
| 13 | G175W | GGACTATGAAATTGACTGGGGGACCATGCTGATC | GATCAGCATGGTCCCCCAGTCAATTTCATAGTCC |
| 14 | V188M | GGCTGCGCTGTGGCTATGAACATCATAATGGGG | CCCCATTATGATGTTCATAGCCACAGCGCAGCC |
| 15 | I190T | TGCGCTGTGGCTGTGAACACTATAATGGGGTTGACC | GGTCAACCCCATTATAGTGTTCACAGCCACAGCGCA |
| 16 | E213del | GGCACCACCAACCAGCAGGAGAACCCCAGCGTCCGA | TCGGACGCTGGGGTTCTCCTGCTGGTTGGTGGTGCC |
| 17 | N214K | AACCAGCAGGAGGAGAAGCCCAGCGTCCGAG | CTCGGACGCTGGGCTTCTCCTCCTGCTGGTT |
| 18 | R218Q | GGAGAACCCCAGCGTCCAGGCTGCCTTCATCCATGTG | CACATGGATGAAGGCAGCCTGGACGCTGGGGTTCTCC |
| 19 | G226S | CATCCATGTGATCAGCGACTTTATGCAGAGCATG | CATGCTCTGCATAAAGTCGCTGATCACATGGATG |
| 20 | S231T | GGCGACTTTATGCAGACCATGGGTGTCCTAGTG | CACTAGGACACCCATGGTCTGCATAAAGTCGCC |
| 21 | G233D | TTTATGCAGAGCATGGATGTCCTAGTGGCAGCC | GGCTGCCACTAGGACATCCATGCTCTGCATAAA |
| 22 | G233R | TTTATGCAGAGCATGCGTGTCCTAGTGGCAGCC | GGCTGCCACTAGGACACGCATGCTCTGCATAAA |
| 23 | P245R | TACTTCAAGCGAGAATACAAGTATGTAGACCCCATCTGC | GCAGATGGGGTCTACATACTTGTATTCTCGCTTGAAGTA |
| 24 | E246K | TACTTCAAGCCAAAATACAAGTATGTAGACCCCATCTGC | GCAGATGGGGTCTACATACTTGTATTTTGGCTTGAAGTA |
| 25 | I269T | GGACAACCTTGACCACCCTGAGAGATGTGATC | GATCACATCTCTCAGGGTGGTCAAGGTTGTCC |
| 26 | E279K | GATCCTGGTGTTGATGAAAGGGACCCCCAAGGG | CCCTTGGGGGTCCCTTTCATCAACACCAGGATC |
| 27 | G299W | TGCTGCTGTCGTGGGAGTGGGTAGAAGCC | GGCTTCTACCCACTCCCACGACAGCAGC |
| 28 | G299R | CTGCTGTCGGTGGAGCGCGTAGAAGCCCTGCACA | TGTGCAGGGCTTCTACGCGCTCCACCGACAGCAG |
| 29 | V300L | CTGCTGTCGGTGGAAGGATTAGAAGCCCTGCACAGC | GCTGTGCAGGGCTTCTAATCCTTCCACCGACAGCAG |
